# Protein Interactome of Muscle Invasive Bladder Cancer

**DOI:** 10.1371/journal.pone.0116404

**Published:** 2015-01-08

**Authors:** Akshay Bhat, Andreas Heinzel, Bernd Mayer, Paul Perco, Irmgard Mühlberger, Holger Husi, Axel S. Merseburger, Jerome Zoidakis, Antonia Vlahou, Joost P. Schanstra, Harald Mischak, Vera Jankowski

**Affiliations:** 1 Charité-Universitätsmedizin Berlin, Med. Klinik IV, Berlin, Germany; 2 Mosaiques diagnostics GmbH, Hannover, Germany; 3 emergentec biodevelopment GmbH, Vienna, Austria; 4 BHF Glasgow Cardiovascular Research Centre, University of Glasgow, Glasgow, United Kingdom; 5 Department of Urology and Urological Oncology, Hannover Medical School, Hannover, Germany; 6 Biomedical Research Foundation Academy of Athens, Biotechnology Division, Athens, Greece; 7 Institut National de la Santé et de la Recherche Médicale (INSERM), U1048, Institute of Cardiovascular and Metabolic Diseases, Toulouse, France; 8 Université de Toulouse III Paul Sabatier, Toulouse, France; 9 Institute for Molecular Cardiovascular Research (IMCAR), Aachen, Germany; Centro Nacional de Investigaciones Oncológicas (CNIO), SPAIN

## Abstract

Muscle invasive bladder carcinoma is a complex, multifactorial disease caused by disruptions and alterations of several molecular pathways that result in heterogeneous phenotypes and variable disease outcome. Combining this disparate knowledge may offer insights for deciphering relevant molecular processes regarding targeted therapeutic approaches guided by molecular signatures allowing improved phenotype profiling. The aim of the study is to characterize muscle invasive bladder carcinoma on a molecular level by incorporating scientific literature screening and signatures from omics profiling. Public domain omics signatures together with molecular features associated with muscle invasive bladder cancer were derived from literature mining to provide 286 unique protein-coding genes. These were integrated in a protein-interaction network to obtain a molecular functional map of the phenotype. This feature map educated on three novel disease-associated pathways with plausible involvement in bladder cancer, namely Regulation of actin cytoskeleton, Neurotrophin signalling pathway and Endocytosis. Systematic integration approaches allow to study the molecular context of individual features reported as associated with a clinical phenotype and could potentially help to improve the molecular mechanistic description of the disorder.

## Introduction

Bladder cancer (BC) presents with an estimate of 72,570 new cases diagnosed and 15,210 deaths across the United States [1] in the year 2013, clearly demonstrating a need for improved diagnosis and therapy. Bladder cancer is the ninth most frequent malignancy with an approximate ratio of 5:1 with respect to non-muscle invasive versus muscle invasive phenotypes [2]. Major confounders are smoking and other occupational exposures along with genetic predispositions, such as e.g. N-acetyltransferase 1 (NAT1), N-acetyltransferase 2 (NAT2) and glutathione S-transferase µ1 (GSTM1) polymorphisms [3]. Though variable for bladder cancer patients, initial symptoms include haematuria and flank pain, commonly represented during advanced cancer stages caused by ureteric obstructions due to invasion of the bladder muscular wall or ureter, together with recurrent urinary tract infections [4, 5]. Evidence suggests that malignant transformation of the bladder is multifactorial and a multitude of genes are involved in the development of muscle invasive or non-muscle invasive phenotype [6, 7]. The major histological type is transitional cell carcinoma occurring in approximately 90% of diagnosed bladder tumours (with the rest being mainly squamous cell carcinomas and adenocarcinomas), with categories of non-invasive papillary (Ta) or flat (Tis), subepithelial invasive (T1), muscle invasive (T2–T4) and metastatic (N+, M+) diseases, all differing in biology, progression characteristics and hence clinical management. Majority of the cases are non-muscle invasive (Tis, Ta, T1) and 10–15% are muscle-invasive tumours (T2–T4), with the latter associated with fast recurrence and poorer prognosis based on progressing towards metastasis formation.

Cystoscopy is the gold standard with a reported sensitivity and specificity in the range of 62–84% and 43–98%, respectively [8]. Due to the invasive nature of the procedure, but also for adding accuracy in the detection, biomarkers assessed in blood or urine are considered as beneficial for supporting clinical assessment [9]. This is also relevant for disease prognosis as biomarkers measured at the DNA, RNA and/or protein levels provide the potential to choose best surveillance measures and treatment regimens for specific patient populations regarding halting the development of muscle invasive disease [10]. Treatment of papillary and non-muscle invasive high-grade carcinoma involves endoscopic transurethral resection of visible tumours followed by adjuvant treatment with intravesical instillation therapy (Mitomycin/Epirubicin or Bacillus Calmette-Guerin (BCG)) depending on the estimated risk for progression. Irrespective of aggressive treatment and vigorous follow-up, 70% of these tumours recur, and 25% of high-grade non-muscle invasive cancers progress into invasive phenotypes [2, 11].

The comparison of the genetic characteristics of muscle-invasive and non-invasive tumours revealed that non-invasive tumours over-express HRAS and FGFR3 or produce highly activated forms of these proteins. As a result, the Ras/MAPK pathways are up-regulated in non-invasive tumours [12]. Muscle-invasive BC is associated with alterations of p53, retinoblastoma protein (RB1) and tumour suppressors controlling cell cycle processes, in addition to elevated expressions in epidermal growth factor receptor (EGFR), human epidermal growth factor receptor 2 (HER2/ErbB2), matrix metallopeptidase 2 (MMP2) and MMP9 and deletions in p16Ink4a and P15Ink4b [3].

High-throughput experimental platform technologies ranging from genomic sequencing to proteomic and metabolomic profiling are now being used for molecular characterization of clinical phenotypes [13–19]. A variety of datasets have become available e.g. in Array Express/Gene Expression Omnibus (GEO) for transcriptomics, Human Proteinpedia for proteomics, or in large data consolidation platforms such as GeneCards [20]. In regard to disease specific omics data, valuable general sources in oncology include TCGA (http://cancergenome.nih.gov/), Oncomine [21], and OMIM [22]. Though omics profiling has provided an abundance of data, technical boundaries involving incompleteness of the individual molecular catalogues together with the static representation of cellular activity limits the insights on molecular processes and their interaction dynamics [23–25]. Despite these challenges, omics-based profiling has significantly advanced bladder cancer research, providing the basis for an integrative analysis approach in delineating a more comprehensive overview of molecular processes and pathways that characterize variations of muscle-invasive urothelial carcinoma [12].

On the effector level, proteins interact and co-operatively form specific molecular processes and pathways. Intermolecular interactions include various types being represented as networks (graphs) with molecular features denoted as nodes (vertices) together with their interactions (edges). A large number of biological pathway resources has become available, including KEGG [26], PANTHER [27], REACTOME [28] and AmiGO [29] described in PathGuide (http://www.pathguide.org/), all displaying well-defined human molecular metabolic and signalling pathways together with disease-specific pathways (e.g. pathways in cancer). Molecular features being identified as associated with bladder cancer can be interpreted on the level of such pathways, adding to a functional interpretation of molecular feature sets characterizing the phenotype.

To add to our understanding of muscle-invasive bladder carcinoma (MIBC), we derived a phenotype-specific network model (interactome) by integrating omics signatures characterizing MIBC, reported in scientific literature and databases. Our procedure incorporated scientific literature screening and signatures from omics profiling, resulting in 1,054 protein-coding genes being associated with MIBC, further consolidating to 286 genes on the interactome level. The results display deriving a systems-level model for molecular phenotyping of bladder cancer muscle invasion, presented as multiple affected pathways.

## Materials and Methods

### Data sources for characterizing bladder cancer pathophysiology

**Figure 1:**
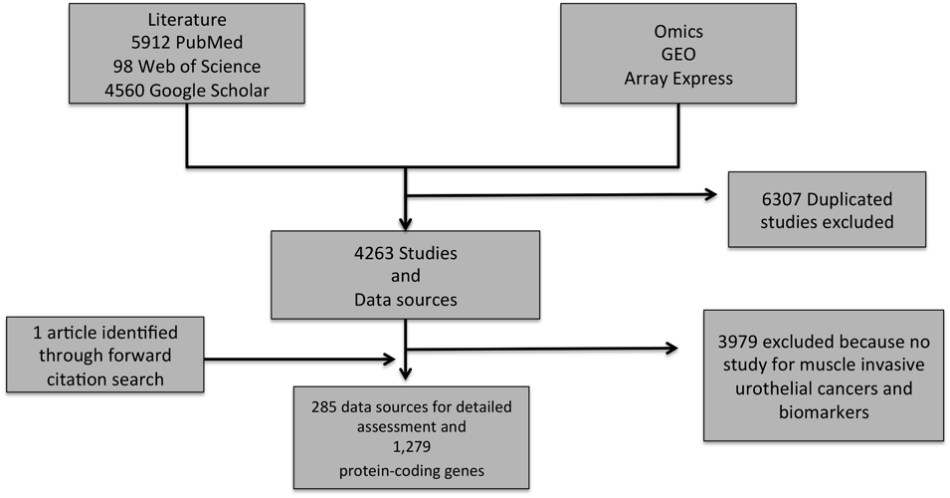
Data assembly workflow. PubMed, Google Scholar and Web of Science literature analysis and Omics data source screening with focus on transcriptomics. From the 4263 abstracts screened 3979 articles were excluded not specifically focusing on muscle-invasive bladder cancer phenotype (stages T2–T4). 188 studies out of 285 articles were discarded, as these did not meet required study designs and 2-fold change in magnitude of differential abundance of identified features. This restriction resulted in 1,279 protein-coding genes and was further used in the systems based analysis for MIBC.

**Figure 2:**
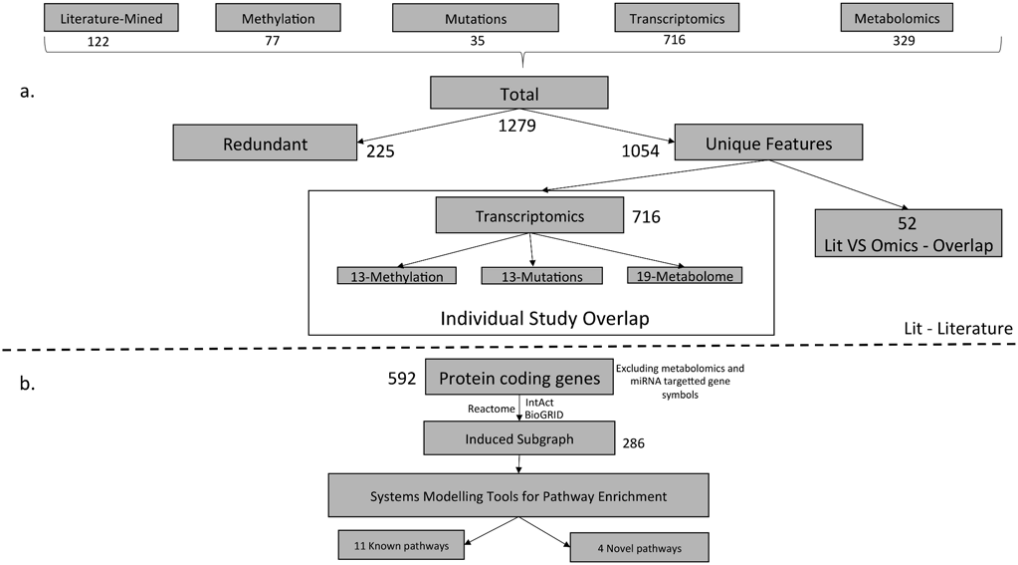
Feature set Overlap. A. Redundant features were discarded from 1,279 protein coding genes resulting in 1,054 unique features.The overlap between individual omics studies and literature were calculated. B. The 1,054 protein coding genes were further reduced to 592 by discarding enzymes linked to metabolites as well as miRNA targeted gene symbols, further included for deriving the induced MIBC subgraph resting on BioGRID, IntAct and Reactome protein interaction information.

For consolidating molecular features associated with muscle invasive bladder cancer, NCBI PubMed, Web of Science, Google Scholar and the omics repositories Gene Expression Omnibus (GEO) [30] and ArrayExpress [31] were queried. The keywords for the literature search included “bladder OR urothelial OR transitional cell” AND “neoplasm OR tumor OR carcinoma” AND “muscle” AND “invas* OR aggress* OR progress* OR inflammation” (Database version of April, 2014). By construction this search query focused specifically on muscle invasive bladder neoplasm. For extracting protein-coding genes associated with these publications gene-2-pubmed as provided by NCBI was used [32]. The list of publications relevant to bladder cancer muscle invasion was isolated from the complete list of papers indexed in PubMed along with the associated gene IDs (ftp://ftp.ncbi.nlm.nih.gov/gene/DATA/gene2pubmed.gz). Profiling experiments were further screened for adequacy in sample size (at least 50 samples included in study design), magnitude of differential abundance (>2-fold change) and the specific phenotypic conditions; T1, T2_a/b_, T3_a/b_, T4_a/b_ (Figs. 1 and 2). In addition, only papers mentioning the keywords “molecular” and “biomarker” were retained for deriving the literature mined MIBC molecules and pathways.

### Interaction data and induced subgraph

Protein interaction information was obtained by querying IntAct [33], BioGRID [34], and Reactome [28] leading to a total of 233,794 interactions covering 13,907 protein-coding genes within the human interactome (Databases in version of April, 2014). Mapping the MIBC associated molecular features on this consolidated interaction network [13] provided an MIBC-specific induced subgraph. MIBC associated features not connected to at least another such feature were disregarded from further analysis.

### Functional analysis

Cytoscape’s plug-ins ClueGO and CluePedia was used to identify pathways that are being over-represented in the set of features located in the induced subgraph [35, 36]. KEGG pathway terms served as the clustering criterion using a two-sided hypergeometry test followed by Bonferroni correction (significance level of 0.05) for identifying significantly affected pathways. General disease pathways (such as pathways in cancer, miRNA’s in cancer, bladder cancer etc.) were discarded to obtain a set of generic pathway terms [13].

### Protein coding gene selection based on literature mining

From the set of MIBC-associated protein-coding genes, each gene symbol was evaluated for being a member of the MIBC pathway set. The evidence of identified pathways and extracted genes involved in MIBC was assessed based on the level of annotation depth, defined as the number of individual studies identifying such protein-coding genes as involved in MIBC. Specifically, such evidence was derived from metadata available in PubMed. Gene-2-pubmed was used for linking the molecules contained in the induced subgraph to publications relevant to bladder cancer muscle invasion. The quality of publications obtained for each molecule was assessed based on manual reviewing. Only papers where a direct link of the molecule to bladder cancer muscle invasion was proven were retained. For the entire pathway set, the ratio between the number of molecules being linked to at least one urinary bladder neoplasm publication and the number of features in the pathway was computed and used for relevance ranking. For individual protein-coding genes identified in literature the number of linked urinary bladder neoplasm publications was used as relevance ranking criterion.

## Results

### Data Mining

Mining of published articles and omics repositories led to a collection of 285 references after manual screening (Fig. 1). This screening was performed to discard duplicated studies retrieved from the varying repositories as well as articles not explicitly focusing on muscle-invasive bladder carcinoma. All molecular features were converted to their official gene symbol by using the UniProt ID Mapping service [37]. The resulting set of references yielded in total 1,279 proteins of which 1,054 were unique protein-coding genes associated with MIBC (S1 Table). For collecting specifically proteins involved in MIBC, we further screened these 285 articles with the keywords (“molecular and biomarker”) to retrieve 122 proteins that had a tag “biomarker” mentioned in these articles (S2 Table). This restriction helped in discarding general articles containing gene symbols that were not associated to the muscle-invasive phenotype. The same set of 285 articles was used to collect all pathways connected to bladder carcinoma. Thus, 11 pathways reported in the literature to be associated with bladder cancer were obtained (S3 Table).

The largest number of features associated with MIBC resulted from transcriptomics with a total of 716 gene symbols. Metabolites were mapped to protein coding genes using the Human Metabolome Database (HMDB) [38] and provided 329 gene symbols. The miRNAs from the transcriptomics studies were mapped to their respective gene targets using the service from miRbase [39, 40]. In addition, DNA-methylation studies provided 77 gene symbols respectively. DNA-mutation studies reported 35 gene symbols. Scientific literature analysis provided 178 protein-coding genes, of which 122 were further annotated as indicators of muscle-invasive bladder cancer. The detailed information on all differentially expressed molecular features is available in S2 and S4–S8 Tables.

### Data Source Overlap

With respect to the feature set overlap, 52 gene symbols were identified in both, literature mining and individual omics signatures (S1 Table). Of the 1,054 unique gene symbols, 716 protein-coding genes were from transcriptomics studies, 13 of these were found on DNA-methylation level, 13 on DNA-mutation level, and 19 on the metabolome level (Fig. 2a). This relatively weak overlap on the level of individual features, however, is a frequent finding in cross-Omics data consolidation, in part stemming from constraints of applied methods, and different sample matrices under investigation in each case [41, 42].

### Induced Subgraph

To increase evidence in regard to the association of molecular features with MIBC we included protein-interaction information as a filter mechanism, i.e. combining evidence from statistical analysis with biological (interaction) data. For this analysis, the molecular feature set was reduced from 1,054 unique protein coding genes to 592 gene symbols. The reduction in the protein list was mainly caused because the protein coding genes indirectly linked from metabolomics and miRNA profiling were not incorporated in the pathway analysis due to low evidence linking to respective targets and enzymes. Further disregarding features not showing interactions to other members of the MIBC set resulted in 286 protein-coding genes represented on the muscle invasive bladder cancer-specific subgraph. The list of the initial 1,054 proteins, the 592 proteins disregarding metabolomics data and miRNA screens, and the list of 286 proteins that form protein-protein interactions in the MIBC subgraph is available in S9 Table. This set of protein-coding genes with strong evidence regarding association with MIBC and holding interactions to other such features was included in pathway analysis (Fig. 2b).

### Pathway enrichment

**Figure 3:**
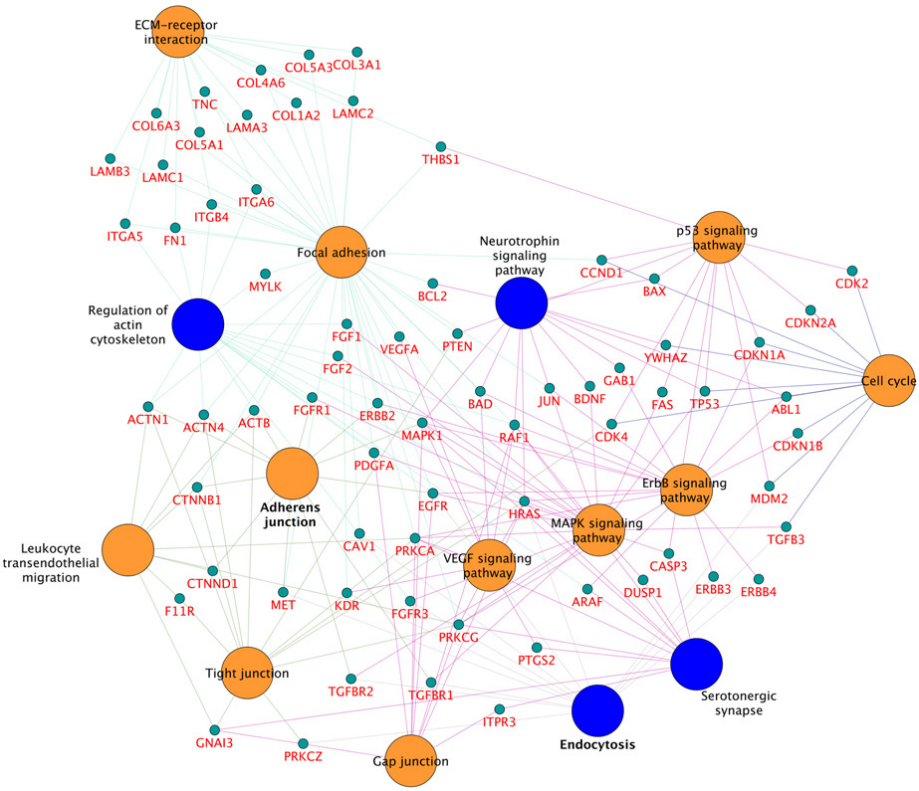
Muscle Invasive Bladder carcinoma interactome, set of 286 protein coding genes. Nodes in orange denote pathways identified as relevant in both literature and enrichment analysis, nodes in blue depicts pathways of relevance according to enrichment analysis. Node size scales with the number of gene symbols encoded in each pathway term.

KEGG pathway enrichment analysis of the MIBC molecular feature set represented on the induced subgraph utilizing ClueGO and CluePedia resulted in 15 molecular pathways being significantly affected in the context of muscle invasive bladder carcinoma (Fig. 3, S3 Table). In detail, 11 of these pathways were previously identified in literature, in addition to 4 presumably novel pathways that resulted from the interactome analysis.

The network in Fig. 3 represents each pathway as individual node, while the edges between pathways denote an approximation of biological interaction between the pathways based on the cross-pathway feature overlap. This pathway map allowed evaluating the functional context of the 122 literature-mined protein candidates in the context of MIBC.

Fig. 3 describes pathway terms enriched using the MIBC-specific induced subgraph. Categorizing the pathway terms in known and novel pathways according to literature, we obtained 11 pathways that were reported in the literature, namely Focal adhesion consisting of 40 protein coding genes, MAPK signalling pathway with 26, ECM-receptor interaction and Cell cycle with 17 features each, p53 with 16, Tight junction and Adherens junction with 15 features each, Leukocyte transendothelial migration with 12, VEGF signalling pathway with 11, and Gap junction containing 10 protein coding genes (see S3 Table). The novel set of pathways that were enriched in the analysis contained 4 pathway terms of which 3 pathways were resting on significant association with the muscle-invasive bladder cancer phenotype, namely Regulation of actin cytoskeleton holding 18 protein coding genes, Endocytosis with 16 and Neurotrophin signalling with 13 (Table 1). The highest overlap in gene symbols was found between regulation of actin cytoskeleton pathway and serotonergic synapse containing ARAF, HRAS, RAF1 and MAPK1, neurotrophin signalling pathway and regulation of actin cytoskeleton pathway, containing MAPK1, RAF1 and HRAS. The overlap of gene symbols between endocytosis and regulation of actin cytoskeleton pathway was FGFR3, EGFR and HRAS, while those between neurotrophin signalling pathway and serotonergic synapse were HRAS, RAF1 and MAPK1. The least overlap of gene symbols between pathways was seen for neurotrophin signalling pathway and endocytosis, only sharing HRAS. Subsequently, there was no protein-coding gene overlapping for endocytosis and serotonergic synapse.

**Table 1:** KEGG pathways significantly associated with muscle invasive bladder carcinoma utilizing the gene set embedded in the induced subgraph.

**KEGG Pathway Name**	**Number of MIBC features**	**Bonferroni corrected p-value**	**Overlapping protein-coding genes**
Regulation of actin cytoskeleton	18	0.005874	**PDGFA, FGF1, RAF1, EGFR, ACTN4, FGFR1, ITGB4, FGFR3, MYLK, HRAS, ACTN1, FGF2, ITGA5, ARAF, FN1, MAPK1, ACTB, ITGA6**
Endocytosis	16	0.0344	**EGFR, MDM2, TGFBR2, FGFR3, HRAS, ERBB3, TGFB3, TGFBR1, ERBB4, CAV1, MET, PRKCZ, KDR**
Neurotrophin signalling pathway	13	0.01022	**BDNF, RAF1, BAD, HRAS, ABL1, GAB1, BCL2, TP53, BAX, YWHAZ, JUN, MAPK1**
Serotonergic synapse	12	0.0278	**RAF1, GNAI3, PRKCG, ITPR3, HRAS, CASP3, PTGS2, DUSP1, ARAF, MAPK1, PRKCA**

Pathway terms, total number of MIBC features associated with the term, Bonferroni corrected p-value and specific gene symbols found overlapping amongst the 4 pathway terms.

**Figure 4:**
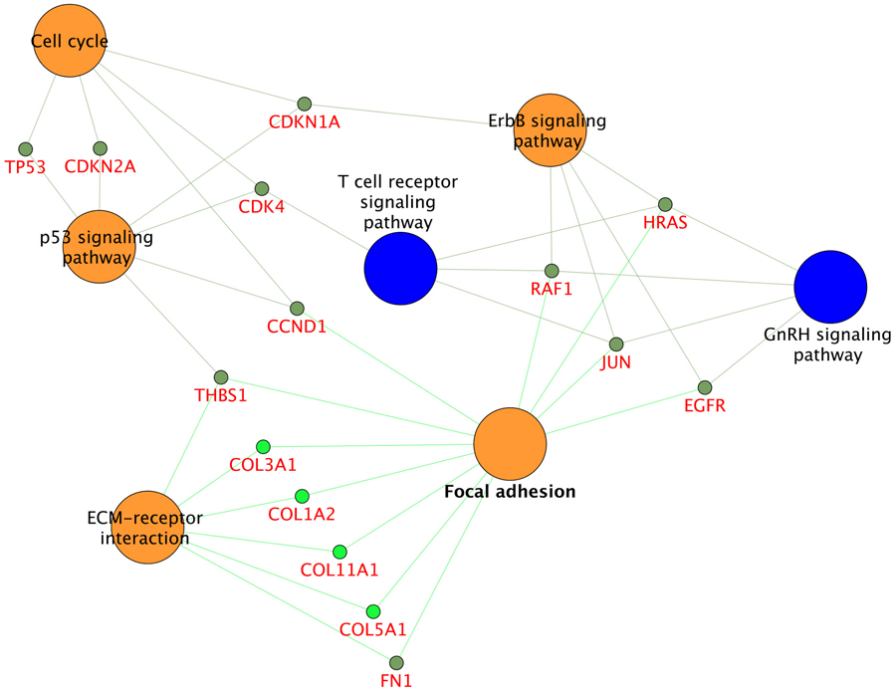
Muscle Invasive Bladder carcinoma pathway enrichment, set of 72 protein coding genes. Nodes in orange denote pathways identified as relevant in both literature and enrichment analysis; nodes in blue depict pathways of relevance according to enrichment analysis. The size of each node size scales with the number of gene symbols encoded in each pathway term.

We performed an additional pathway enrichment analysis that involved an alternative set of gene symbols. From the full set of protein coding genes (707 molecules excluding metabolite and miRNA targetted gene symbols), we restricted to members being present in more than one study type (e.g. ERBB2 was found in proteomics, mRNA and literature mining). This restriction resulted in 72 gene symbols, again forwarded to pathway enrichment analysis. Fig. 4 details pathway terms enriched using this reduced set of protein coding genes. Seven pathway terms were enriched in this analysis. Categorizing these pathway terms into known from literature and novel pathways, 5 pathway terms were reported in literature and 2 pathways were novel findings. The 5 previously known pathway terms included Focal adhesion, Cell cycle, and p53 signalling pathway, ECM-receptor interaction, and ErbB signalling (S10 Table). In regard to the 2 novel pathways that were enriched from this analysis, the pathway terms were T cell receptor signaling pathway and GnRH signaling. Table 2 details all pathways with their overlapping gene symbols that were retrieved from this analysis. Regarding pathways with literature evidence the enrichment resting on the 72 gene symbols had a full overlap with the 15 pathway terms retrieved using the induced subgraph MIBC feature set. The 2 pathways not showing an overlap were the novel pathways resting on the second gene set namely T cell receptor signalling and GnRH signalling (Fig. 4, S3 and S10 Tables).

**Table 2:** KEGG pathways significantly associated with MIBC according to gene symbols found in more than one omics study type.

**KEGG Pathway Name**	**Number of features**	**Bonferroni corrected p-value**	**Overlapping protein-coding genes**
Focal adhesion	16	2.31E-011	**COL3A1, HRAS, CCND1, COL11A1, FN1, THBS1, JUN, COL5A1, RAF1, EGFR, COL1A2**
Cell cycle	7	0.00141	**CCND1, TP53, CDK4, CDKN2A, CDKN1A**
p53 signaling pathway	6	4.17E-04	**CCND1, THBS1, TP53, CDK4, CDKN2A, CDKN1A**
ErbB signalling pathway	6	0.00172	**HRAS, JUN, RAF1, CDKN1A, EGFR**
ECM-receptor interaction	6	0.00151	**COL3A1, COL11A1, FN1, THBS1, COL5A1, COL1A2**
GnRH signalling pathway	5	0.0360	**HRAS, JUN, RAF1, EGFR**
T cell receptor signalling pathway	5	0.048	**HRAS, JUN, CDK4, RAF1**

Pathway terms, number of molecules associated with the term, Bonferroni corrected p-value and specific gene symbols found overlapping amongst the 7 pathway terms.

## Discussion

Understanding the molecular pathophysiology of muscle-invasive bladder carcinoma and revealing the network of pathways involved in muscle invasion could lead to targeted therapy. In addition, addressing specific dys-regulated pathways linked to progressive disease holds the promise of supporting an improved, biomarker-based risk assessment followed by stratified clinical intervention [2]. High throughput screening platforms have provided a wealth of information in describing the molecular status reflecting a clinical phenotype, including bladder carcinoma [43, 44]. Experiments based on expression profiling using microarrays, and fractionation techniques coupled to mass spectrometry utilizing tissue and urine as sample matrix have supported molecular pathway-based discovery in bladder muscle invasive neoplasms [12, 45]. The present study intended to characterize muscle invasive bladder carcinoma by incorporating scientific literature screening and signatures from omics profiling further linked in an interaction context, resulting in a set of 286 protein-coding genes. Such analysis on the level of networks and pathways was chosen with the expectation that miscellaneously found phenotypic features consolidate on a pathway level, under the assumption that they are functionally linked and collectively affect the disease phenotype.

High-throughput DNA sequencing can yield erroneous data [46]. MS based proteomics experiments generate enormous datasets that need to be carefully assessed [47].

Biological pathway databases play an essential role in annotating protein-coding genes resulting from high-throughput profiling approaches. There are approximately 547 pathway database resources available as listed in PathGuide (http://www.pathguide.org/). Albeit there are several well curated and reliable pathway database resources as also described by our group [48], significant efforts have been taken to expand biological pathway coverage beyond any single pathway data source. This is frequently carried out by integrating different sources in order to build high quality integrative pathway models without sacrificing data quality. However, biological data integration from heterogeneous sources has been challenging due to variability at the syntactic and semantic level. Syntactic variability is due to heterogeneity of molecular feature and pathway data formats, representation schemas and retrieval methods. Semantic variability is due to incompatible pathway names, signalling event representations and molecular identifiers. For example, different pathway databases may choose to provide information on post-translation modifications, interacting proteins within a complex, or cellular location. Hence all these limitations have inhibited the growth of high quality integrative pathway models [49–51].

Another issue that arises when aiming to integrate data from different omics platforms is that conflicting results can be obtained. For example in some muscle invasive tumours presented in [52], transcriptomics analysis proved that the mRNA level of EGFR is up-regulated, whereas proteomic analysis did not show differential expression at the protein level. One explanation for such discrepancy may be translational regulation.

In KEGG, biological pathway categorization is currently available for several human key cellular processes [13]. Mapping MIBC-specific features (corrected on the level of the induced subgraph utilizing protein interaction information) to KEGG and performing enrichment analysis provided a total of 15 pathways (4 novel and 11 cited in published studies). 68 of 122 literature-mined protein candidates of relevance in muscle-invasive bladder cancer were identified as members of the identified pathways. This enabled to comprehensively rank pathways allowing the shortlisting of terms being individually discussed in the specific context of MIBC.

We focused on expanding our knowledge on muscle invasive urothelial neoplasm affected at the molecular level by comprehensively mapping available molecular datasets to pathways to build an interactome network utilizing public domain data sources. By differentiating the pathways based on previously described pathways and novel ones we obtained 11 modules that were known in context of bladder cancer muscle-invasion and 4 novel pathways. In respect to the previously known urothelial bladder muscle-invasive carcinoma pathways, our analysis retrieved pathways such as MAPK signalling pathway, ErbB signalling pathway, cell-cycle pathways and VEGF signalling pathway, hence confirming the systems-level approach for the particular phenotype [3, 12, 53, 54].

On the other hand, the interactome results also retrieved significant pathways comprising of signalling pathways, cytoskeleton remodelling pathways and neuromuscular junctions. Three molecular pathways were highly significant from the analysis, namely regulation of actin cytoskeleton, neurotrophin signalling pathway and endocytosis.

Neurotrophins are a class of closely related proteins that control the function, survival and development of neurons and have the potential to activate tropomyosin-related kinase (Trk) family of receptors and down regulate tumour necrosis factor superfamily (p75NTR) through which PI3K/Akt, Ras/Raf/MAP kinase, NF-kappa B and Jun kinase signalling pathways are triggered. Trk-receptors with neurotrophin ligands have been identified as initiating tumour progression, and the signalling pathway neurotrophins-Trk has been reported as a target for therapeutic intervention in hormone-refractory prostate cancer (HRPC) and in human astrocytomas, and potentially could play a role in urothelial carcinoma [55–58]. Endocytic pathways represent multiple aberrations in human neoplasms by being tightly and bi-directionally connected to signalling pathways that could indicate malignant transformations of the tumours. One of its regulators, DAB2, has also been reported to be prominent in advanced stages of urothelial cancers, where a decreased expression of the molecule could be observed in metastatic stages, and has been associated with high probabilities of recurrence and bladder carcinoma mortality [59–61]. Deregulation of actin bound proteins, namely p38β, ATF3 and Rho family of small GTPases which are involved in cytoskeletal remodelling, causes aberrant cell motility that leads to the muscle-invasive and metastatic phenotypes in cancer [62–65]. Our analysis highlights the role of the cytoskeletal remodelling pathway that contains integrins, cadherins and adhesion proteins. The respective molecular pathways discussed above open new avenues for further investigation of urothelial muscle-invasive carcinoma. One enriched pathway that did not show any direct relation to bladder cancer was serotonergic synapse that contained 12 protein molecules (S3 Table).

The bioinformatics approach reported here involved integrating available public domain data sets in context of bladder muscle-invasive carcinoma on an interaction network, and further mapping them to biological pathway sources to reveal 15 pathways as being affected in progressive disease. Eleven from these pathways were discussed previously in the context of MIBC. It should be taken into account that while using such computational techniques to integrate molecular signatures from varying resources, certain technical issues regarding the use of appropriate global identifier need to be considered. In our approach, we discarded metabolite and micro-RNA targets for the pathway enrichment analysis (i.e. gene symbols mapped from metabolomics and miRNA data, service provided by HMDB and miRBase), resulting in 592 features from the total of 1,054 protein coding genes. This is mainly driven by hampered translation of metabolite and microRNA profiles to the level of involved protein coding genes, be it on the target or enzyme level. In regard to genomics and epigenetics, we only incorporated those gene symbols that contained epigenetic information on the protein/mRNA abundance levels for the interactome analysis.

On the other hand, the two pathway terms GnHR receptor and T cell receptor signalling pathways found as enriched on the basis of the 72 gene symbols being multiply identified in, were not retrieved from the analysis resting on the full set of 286 features being derived from the induced subgraph. Data evidence and selection biases clearly affect results of such integrated analysis demanding strict quality control of input data sets as followed in our study.

Apparently, each individual functional context highlights specific aspects of bladder cancer pathophysiology, but only providing limited characterization of clinical outcome on the cohort level.

In summary, automated data retrieval from the literature resulted in a first collection of molecular features associated with MIBC, and, complementing with omics profiling data, allowed augmenting a mechanistic (pathway) map linked to MIBC. From the cross-sectional nature of the underlying molecular feature collection no direct conclusion can be drawn regarding the prognostic relevance of individual pathways. However, deriving bladder cancer-associated protein coding genes on the basis of such pathway maps provides a systematic foundation for experimental analysis regarding association with development of muscle-invasive disease. We are confident that this approach can form the basis to rational selection of biomarkers for enabling targeted analysis of potentially relevant key molecules.

## Conclusions

Our results suggest that there is a complex interplay between interacting pathways that characterizes the muscle invasive phenotype of invasive bladder cancer. We developed an integrated molecular model of muscle invasive bladder cancer to allow selecting protein-coding genes on the pathway level aimed at capturing a set of pathways of potential relevance in tumour progression. Further experimental validation of Neurotrophin signalling pathway, Regulation of actin cytoskeleton and Endocytosis with respect to disease progression and treatment response in muscle-invasive bladder carcinoma is indicated.

## Supporting Information

S1 Table Overlap Analysis, molecular feature sets from literature mining and omics screening. Sheet one lists protein coding genes retrieved from DNA mutation, methylation, transcriptomics, metabolomics and literature screening. Redundant entries were ranked based on the frequency of occurence. Combing all protein coding genes resulted in 1,054 unique protein coding genes.
(XLS)

S2 Table Data Inclusion for Muscle Invasive Bladder Carcinoma—Non-Redundant Features from Literature Mining. Provided are gene symbols pertaining to the muscle-invasive phenotype (T2–T4) of bladder carcinoma identified in scientific literature, together with expression levels and PubMed identifiers.
(XLS)

S3 Table Enriched Pathways in context of Urothelial Muscle-Invasive Carcinoma using the 286 molecular features from the induced subgraph. Provided are KEGG pathways together with the number of MIBC-associated features, overlapping literature-mined gene symbols and Bonferonni corrected p-values. The supporting table is divided into two sheets namely Novel pathways and Literature-known pathways.
(XLS)

S4 Table Data Inclusion for Muscle-Invasive Bladder Carcinoma—DNA-Methylation. Provided are gene symbols pertaining to the muscle-invasive phenotype (T2–T4) of bladder carcinoma identified from methylation studies, together with expression and methylation levels in addition to their PubMed identifiers.
(XLSX)

S5 Table Data Inclusion for Muscle-Invasive Bladder Carcinoma—mRNA. Provided are gene symbols pertaining to the muscle-invasive phenotype (T2–T4) of bladder carcinoma resulting from transcriptomics studies (inculsion criteria of >50 molecules in the study), together with expression levels and PubMed identifiers. Protein coding genes that only hold differential expression information are provided with links from available studies.
(XLS)

S6 Table Data Inclusion for Muscle-Invasive Bladder Carcinoma—miRNA. Provided are miRNA targets pertaining to the muscle-invasive phenotype (T2–T4) of bladder carcinoma from transcriptomics studies (inculsion criteria of >50 molecules in the study), together with expression levels and PubMed identifiers.
(XLS)

S7 Table Data Inclusion for Muscle-Invasive Bladder Carcinoma—Metabolites. Provided are underlying enzymes for metabolites pertaining to the muscle-invasive phenotype (T2–T4) of bladder carcinoma from metabolomics studies (inculsion criteria of >50 molecules in the study), together with expression levels and PubMed identifiers. Protein coding genes that only hold differential expression information are provided with links to the data retrieved from available studies.
(XLS)

S8 Table Data Inclusion for Muscle-Invasive Bladder Carcinoma—DNA Mutation. Provided are protein coding genes with significant levels of mutations pertaining to muscle-invasive phenotype (T2–T4) of bladder carcinoma and PubMed identifiers.
(XLS)

S9 Table Protein coding genes involved in the induced Subgraph. Provided are the MIBC-asociated gene symbols in three different columns; a. 1,054 unique gene symbols from initial consolidation, b. 592 gene symbols excluding enzymes from metabolite signatures as well as miRNA targets c. 286 gene symbols that formed the induced subgraph, and d. interaction information among the 286 gene symbols.
(XLS)

S10 Table Enriched Pathways in context of Urothelial Muscle-Invasive Carcinoma using the 72 molecules that were found in more than one omics study. 7 pathway terms were enriched. The set of protein coding genes included is listed in sheet 2. Sheet 3 and 4 list known and novel pathways together with the number of protein coding genes assigned and Bonferroni corrected p-values. Sheet 5 and 6 list the overlapping gene symbols for all identified pathways.
(XLS)
